# *Staphylococcus hominis*: a rare cause of endophthalmitis

**DOI:** 10.5935/0004-2749.20230035

**Published:** 2023

**Authors:** Aslan Aykut, Mehmet Orkun Sevik, Burçin Şan, Özlem Şahin

**Affiliations:** 1 Department of Ophthalmology, Marmara University School of Medicine, Istanbul, Turkey.

**Keywords:** Endophthalmitis, Eye infection, bacterial, Staphylococcus hominis/isolation & purification, Bevacizumab, Intravitreal injection, Humans, Case report, Endoftalmite, Infecç**ão** ocular bacteriana, Staphylococcus hominis/isolamento & purificação, Bevacizumab, Injeção intravítrea, Humanos, Relato de caso

## Abstract

*Staphylococcus hominis (S. hominis)* is a coagulase-negative Staphylococci and an infrequent cause of endophthalmitis. Due to its ability to produce biofilm, especially in diabetic patients, strains may acquire antibiotic resistance. We present two cases of *S. hominis* endophthalmitis, one with acute endophthalmitis after intravitreal bevacizumab injection and one with chronic endophthalmitis following undiagnosed penetrating ocular trauma. Although there are only four published *S. hominis* endophthalmitis cases in the literature, to the best of our knowledge, there has been no previously published case after intravitreal bevacizumab.

## Abstract (pt)

*Staphylococcus hominis (S. hominis)* é um estafilococo coagulase-negativo e uma causa pouco frequente de endoftalmite. Devido à sua capacidade de produzir biofilme, especialmente em pacientes diabéticos, cepas dessa bactéria podem adquirir resistência a antibióticos. Este relato apresenta dois casos de endoftalmite por *S. hominis:* um de endoftalmite aguda após injeção intravítrea de bevacizumabe e outro de endoftalmite crônica após trauma ocular penetrante não diagnosticado. Embora existam apenas quatro casos de endoftalmite por *S. hominis* publicados na literatura, até onde sabemos não houve nenhum caso publicado anteriormente após bevacizumabe intravítreo.

## INTRODUCTION

Coagulase-negative Staphylococci (CoNS) are common inhabitants of the ocular surface^(1)^. They are the most frequently encountered microorganisms from post-procedural endophthalmitis^(2)^. However, *S. hominis*, a member of CoNS, is rarely encountered as a cause of endophthalmitis^(3-6)^. To our knowledge, there are only four cases of S. hominis endophthalmitis in the literature. The first case was described as chronic endophthalmitis after phacoemulsification surgery, presenting with capsular hypopyon^(3)^. The other cases were acute postoperative endophthalmitis after phacoemulsification surgery, chronic endophthalmitis following ocular trauma with a retained intralenticular foreign body, and delayed-onset endophthalmitis after uneventful phacoemulsification surgery^(4-6)^. Here, we present a unique case of acute endophthalmitis after intravitreal bevacizumab injection and a case of chronic endophthalmitis following undiagnosed penetrating ocular trauma, highlighting a possibility of *S. hominis* endophthalmitis in such cases and the need for caution to identify and treat the causative pathogen.

## CASE REPORT

### Case 1

**Figure 1:**
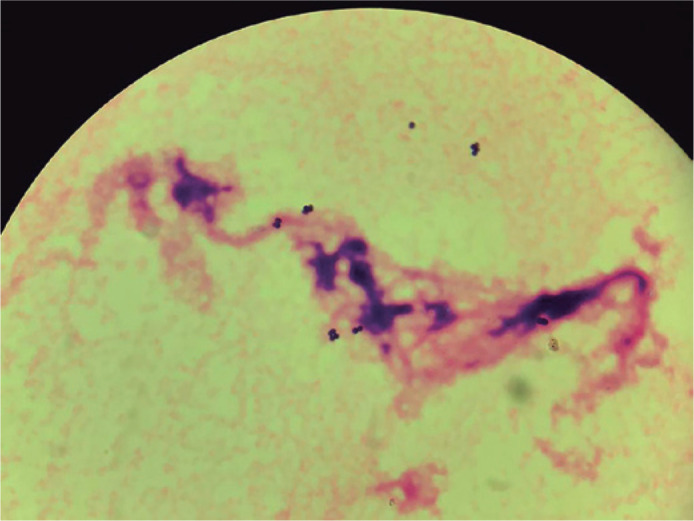
Direct microscopy with Gram staining of the vitreous specimen showing gram-positive cocci.

A 60-year-old female patient presented with the best-corrected visual acuity (BCVA) of counting fingers (CF) at 30 cm with tractional retinal detachment (TRD) in her left eye. Medical history revealed that the patient had insulin-dependent diabetes mellitus (DM) and chronic renal failure for five years. She was previously treated with bilateral pan-retinal photocoagulation for proliferative diabetic retinopathy. For TRD, we planned pars plana vitrectomy (PPV) with intravitreal bevacizumab (IVB) injection three days before the surgery. However, two days after the IVB injection, the patient presented with decreased vision, epiphora, severe pain, and photophobia. Her BCVA was CF at 15 cm with corneal edema, hypopyon (0.7 mm), and dense vitritis in the affected eye. The patient was diagnosed with postinjection endophthalmitis. Samples were taken from aqueous (0.1 ml) and vitreous (0.2 ml) fluids and sent to microbial culture, and simultaneous intravitreal antibiotic (1 mg/0.1 ml vancomycin and 2.25 mg/0.1 ml ceftazidime) injection was administered. Direct microscopy with Gram staining of the aqueous and vitreous specimens showed gram-positive cocci (Figure 1), and methicillin-sensitive *Staphylococcus hominis (S. hominis)* grew in the cultures of both specimens (Table 1). Twelve hours after intravitreal antibiotic injection, the patient’s BCVA dropped to hand motion (HM), and urgent PPV was performed with total vitreous removal, separation of tractions as much as possible, and 5000 cst silicone oil (SiO; Teknomek^®^, Istanbul, Turkey) tamponade. After SiO injection, ten times diluted vancomycin (0.1 mg/ 0.1 ml) was administered.

**Table 1:** Antibiotic Sensitivity of the *Staphylococcus hominis* in Two Cases

Antibiotic	Case 1	Case 2
Erythromycin	S	S
Fosfomycin	R	R
Fusidic Acid	R	R
Gentamycin	S	S
Clindamycin	S	S
Ciprofloxacin	S	S
Penicillin	S	S
Methicillin	S	S
Trimethoprim/Sulfamethoxazole	S	S
Vancomycin	S	S

Sensitivities were determined as the minimum inhibitory concentrations of 80% and 100% for bacteriostatic and bactericidal antibiotics, respectively.

S= sensitive; R= resistant.

One week after the PPV, the BCVA was HM with mild anterior chamber inflammation and intraocular pressure (IOP) of 8 mmHg. At 6 months postoperatively, the BCVA was preserved as HM with an IOP of 6 mmHg under SiO. There was no recurrence of intraocular infection.

### Case 2

An 86-year-old male patient was admitted to our clinic with a gradual decrease of vision and pain after an injury from a bush in his eye 1.5 months ago. The left eye examination revealed BCVA of light perception, 1 × 1 mm corneal scar, 3 + anterior chamber cell, rubeosis iridis, dense corticonuclear cataract, and IOP of 4 mmHg. Dense vitreous condensation was evident on B-scan ultrasonography, and orbital computed tomography showed no foreign body. Urgent PPV with phacoemulsification was planned, suspecting endophthalmitis secondary to penetrating trauma. A tiny anterior capsular tear topographically matching with the corneal scar was noticed during surgery, revealing the cause of the cataract and confirming our diagnosis. Therefore, aqueous and vitreous samples were taken and sent for culture. PPV was completed with 5000 cst SiO (Teknomek^®^, Istanbul, Turkey) tamponade and intravitreal ten times diluted antibiotic injections (0.1 mg/0.1 ml vancomycin and 0.225 mg/0.1 ml ceftazidime). Although direct microscopy with Gram staining showed no mi­croorganisms, methicillin-sensitive *S. hominis* grew in both vitreous and aqueous cultures (Table 1). At 6 months postoperatively, the patient’s BCVA was 20/400 with an IOP of 9 mmHg under SiO. The patient was scheduled for SiO removal.

## DISCUSSION

Biofilm production is one of the main reasons why bacteria in the ocular flora gain virulence^(7)^. With this ability, they can adhere to abiotic (e.g., needles) or biotic (e.g., conjunctiva) surfaces^(7)^. In a study investigating the potential sources of bacterial contamination during intravitreal injections, although the subgroup analysis was not mentioned, CoNS was the most frequently isolated bacteria in the cultures^(8)^. A recent study using culture-dependent and culture-independent approaches found a wide variety of bacteria on intravitreal needles.^(9)^ In this study, 5 of the 18 needles (28%) sent to culture were culture-positive, and one of the three microorganisms causing culture-positivity was *S. hominis*^(9)^.

For CoNS, the rate of biofilm production in the conjunctiva of patients with DM is 30% compared to 10% in patients without DM^(7)^. Also, biofilm production is associated with antibiotic resistance^(7)^. In a recent study, the highest vancomycin resistance rate from biofilm-positive samples was found to belong to *S. hominis* isolates^(7)^. In previously reported *S. hominis* endophthalmitis cases, the microorganism was resistant to vancomycin, ciprofloxacin, moxifloxacin, and cefazolin in the case of acute endophthalmitis after cataract surgery^(4)^, and penicillin in the case of delayed-onset endophthalmitis^(6)^. Antibiotic susceptibility testing was not mentioned in the other two published cases^(3,5)^. Fortunately, considering our reported patients, isolated *S. hominis* was sensitive to vancomycin, so the infection was not refractory to treatment.

CoNS are well-known pathogens of post-traumatic endophthalmitis, and *Staphylococcus epidermidis* and *Staphylococcus saprophyticus* were the prevailing microorganisms in this group^(10)^. There is one reported case of chronic *S. hominis* endophthalmitis in the literature 14 months after the initial trauma with an unre­cognized intralenticular foreign body^(5)^. Like in that case, in our second case, after an undiagnosed penetrating trauma with anterior capsule rupture, chronic endophthalmitis may have developed as a result of the inoculation of *S. hominis* into the avascular lens where it was sequestered and gained virulence. This may explain why there was no acute endophthalmitis manifestation in both cases since there was no direct inoculation of microorganisms into the vitreous.

In conclusion, *S. hominis* may be an overlooked pathogen for endophthalmitis cases, since, to our knowledge, we report the fifth and sixth cases in the literature, one of which is the only case of endophthalmitis occurring after IVB injection in the literature. Due to their ability to develop antibiotic resistance, especially to vancomycin, the most frequently administered empirical antibiotic in endophthalmitis, care should be given to identify the pathogen.
